# Air Pollution and Oral Health in the European Union: Preventive System Capacity as a Structural Modifier of Caries Burden

**DOI:** 10.3390/dj14070440

**Published:** 2026-07-15

**Authors:** Cassandra Lupita, Anca-Cristina Perpelea, Laura-Cristina Rusu, Iulia Muntean, Oana-Ramona Lobonț, Magda-Mihaela Luca

**Affiliations:** 1 Department of Oral Pathology, Multidisciplinary Center for Research, Evaluation, Diagnosis and Therapies in Oral Medicine, “Victor Babes” University of Medicine and Pharmacy Timisoara, 2 Eftimie Murgu Sq., 300041 Timisoara, Romania; 2 Department of Organization, Professional Legislation and Management of the Dental Office, Faculty of Dental Medicine, “Carol Davila” University of Medicine and Pharmacy, 17-23 Plevnei Street, 020021 Bucharest, Romania; 3 Finance, Business Information Systems and Modeling Department, Faculty of Economics and Business Administration, West University of Timisoara, 300115 Timisoara, Romania; 4 Department of Pediatric Dentistry, Faculty of Dental Medicine, “Victor Babes” University of Medicine and Pharmacy Timisoara, Eftimie Murgu Square 2, 300041 Timisoara, Romania

**Keywords:** dental caries, edentulism, preventive dental programs, air pollution, PM2.5, unmet dental care needs, oral health disparities, panel data analysis, European Union

## Abstract

**Background/Objectives:** Environmental air pollution and structural barriers to preventive dental care are increasingly recognized as determinants of population health inequalities. However, their combined association with oral disease burden across European Union (EU) Member States remains insufficiently examined. This study evaluated whether ambient particulate matter (PM2.5) exposure is associated with oral health outcomes and whether preventive system capacity modifies this association. **Methods:** An ecological panel analysis was conducted using country-level data from the 27 EU Member States for the period 2020–2023. Age-standardized prevalence of untreated dental caries (primary outcome), edentulism (secondary outcome), and periodontal diseases (exploratory outcome) were obtained from the Global Burden of Disease database. PM2.5 exposure was operationalized using national mean annual baseline values. Preventive care access was proxied by the percentage of the population reporting unmet dental care needs (Eurostat EU-SILC). Pooled panel regression models with year fixed effects and country-clustered robust standard errors were estimated. An interaction term between PM2.5 exposure and unmet dental care needs was included to assess effect modification. **Results:** PM2.5 exposure was not independently associated with caries prevalence after adjustment for preventive access and GDP per capita. However, a statistically significant interaction was observed: higher PM2.5 levels were associated with increased caries prevalence in countries reporting greater unmet dental care needs. PM2.5 exposure was positively associated with edentulism prevalence, independent of preventive access. No significant associations were detected for periodontal diseases. **Conclusions:** Based on this ecological analysis of European Union Member States, higher PM2.5 exposure in combination with greater unmet dental care needs was associated with higher population-level caries prevalence, whereas PM2.5 exposure alone showed a positive association with edentulism prevalence. These findings are exploratory and should be interpreted with caution, given the use of aggregated country-level data, the potential for ecological fallacy, and the absence of causal inference. Further individual-level and longitudinal studies are needed to clarify these relationships and their underlying mechanisms.

## 1. Introduction

Oral diseases, although involving microbial and biofilm-mediated processes, are classified among the most prevalent non-communicable diseases worldwide due to their chronic nature, multifactorial etiology, shared risk factors, and substantial public health burden [1]. Untreated dental caries represents the most common health condition globally across all age groups, while severe tooth loss and edentulism reflect the cumulative consequences of long-standing oral pathology and structural failures in prevention and timely treatment [2,3,4]. Despite relatively advanced healthcare systems, marked inequalities in oral health outcomes persist across European Union (EU) Member States [5,6].

Dental caries is a multifactorial disease influenced by behavioral, biological, and structural determinants. Established risk factors include high sugar intake, inadequate oral hygiene, reduced fluoride exposure, and socioeconomic disadvantage [7,8,9]. At the population level, structural characteristics of health systems—such as accessibility, affordability, and organization of dental services—play a decisive role in shaping disease trajectories [10]. Preventive dental programs, including fluoride-based interventions, fissure sealant programs, and early screening initiatives, are designed to reduce early-stage disease and limit progression toward tooth loss [11,12]. However, their effectiveness depends not only on formal implementation but also on equitable access and population-level uptake [13].

Limited access to dental services has been associated with delayed diagnosis, higher prevalence of untreated caries, and increased risk of cumulative tooth loss [14,15]. Within the EU, self-reported unmet dental care needs are widely used as a structural indicator of barriers related to financial constraints, geographic inaccessibility, and waiting times [16]. These barriers may weaken preventive system performance and amplify disparities in oral health outcomes.

In parallel, environmental exposure has increasingly been recognized as a determinant of non-communicable diseases. Fine particulate matter (PM2.5), a major component of ambient air pollution, has been linked to systemic inflammation, oxidative stress, and increased risk of cardiovascular and respiratory morbidity [17,18,19]. Emerging evidence suggests that environmental stressors may also affect oral health through inflammatory and immunological pathways [20]. Nevertheless, large-scale comparative analyses integrating environmental exposure and structural access to preventive dental care remain scarce in the European context.

Most existing studies examine either individual-level behavioral risk factors or socioeconomic inequalities without incorporating environmental variables into cross-country frameworks [21]. Consequently, the extent to which environmental exposure is associated with oral disease burden, and whether this contribution depends on preventive system capacity, remains insufficiently explored.

The present study addresses this gap by evaluating the association between ambient PM2.5 exposure, preventive dental care access, and oral health outcomes across EU Member States during 2020–2023 using publicly available longitudinal country-level data. The primary outcome is the prevalence of untreated dental caries, reflecting early-stage disease targeted by preventive strategies, while edentulism is analyzed as a secondary outcome representing cumulative disease progression. We hypothesized that the association between environmental exposure and oral health outcomes may vary according to the preventive care context.

## 2. Materials and Methods

### 2.1. Study Design

This study was designed as an observational, non-interventional ecological panel analysis using country-level data from the 27 European Union (EU) Member States. The unit of analysis was the Member State, with annual observations covering the period 2020–2023, corresponding to the latest year for which harmonized data were simultaneously available across all primary data sources.

The analytical framework was developed to investigate temporal and cross-sectional associations between environmental exposure (ambient PM2.5 concentration), access to preventive dental care (proxied by unmet dental care needs), and population-level oral health outcomes. By incorporating repeated yearly observations for each Member State, the panel structure enabled the simultaneous assessment of:

(i) between-country heterogeneity in oral health burden and structural determinants (e.g., differences in access to dental services, economic development, and healthcare system organization);
(ii) temporal variation in oral health outcomes and preventive access indicators during the study period.

The panel approach facilitates the simultaneous evaluation of cross-country heterogeneity and temporal variation in oral health outcomes and preventive access indicators.

The ecological design was selected due to the availability of harmonized country-level datasets and the policy-oriented objective of evaluating structural determinants of oral health within the EU context. Consequently, findings reflect population-level associations and should not be interpreted as individual-level causal effects.

All data were obtained from publicly accessible international databases and consisted exclusively of aggregated national indicators (e.g., prevalence rates, percentage-based access measures, and mean annual environmental concentrations). No individual-level or identifiable data were used; therefore, ethical approval and informed consent were not required.

### 2.2. Data Sources

Oral health outcome data were obtained from the Global Burden of Disease (GBD) Study 2023 Results Tool, managed by the Institute for Health Metrics and Evaluation (IHME). Data were downloaded through the publicly accessible GBD Results interface (https://vizhub.healthdata.org/gbd-results/ (accessed on 25 November 2025)). The following parameters were selected:

- Measure: Prevalence
- Metric: Rate
- Age: All ages
- Sex: Both sexes
- Location: European Union Member States
- Years: 2020–2023 (latest year consistently available across outcomes)

Three disease categories were extracted:

1. Caries of permanent teeth (primary outcome);
2. Edentulism and severe tooth loss (secondary outcome);
3. Periodontal diseases (exploratory outcome).

Prevalence rates were expressed per 100,000 population and represent age-standardized estimates as provided by the GBD database.

Environmental exposure data were obtained from the World Bank World Development Indicators (WDI) database (https://data.worldbank.org/indicator/EN.ATM.PM25.MC.M3 (accessed on 25 November 2025)).

The indicator “PM2.5 air pollution, mean annual exposure (micrograms per cubic meter)” was selected. Country-level mean annual exposure values (µg/m^3^) were extracted for EU Member States. Because annual PM2.5 exposure data were not consistently available for all EU Member States across the full study period using comparable reporting frameworks, constructing a balanced panel of yearly exposure values aligned with the health outcome data was not feasible.

Given differences in year availability across datasets, PM2.5 exposure was operationalized using the 2020 annual mean value as a baseline environmental indicator applied consistently across the analytical panel.

Preventive dental care access was operationalized using data from Eurostat—European Union Statistics on Income and Living Conditions (EU-SILC). The dataset “Self-reported unmet needs for dental examination or treatment” (dataset code: hlth_silc_08) was accessed via the Eurostat Data Browser (https://ec.europa.eu/eurostat/databrowser/ (accessed on 25 November 2025)).

The indicator represents the percentage of the population reporting unmet dental care needs due to financial, geographical, or waiting-time barriers. Annual country-level percentages were extracted for 2020–2023.

Socioeconomic covariates were obtained from Eurostat databases:

- Gross Domestic Product (GDP) per capita (dataset: nama_10_pc), expressed in euros per inhabitant, as a macroeconomic control variable;
- Dentists per 100,000 inhabitants (dataset: hlth_rs_prs2);
- Educational attainment indicators, where available.

All datasets were downloaded in CSV format from official institutional repositories. Data extraction was performed in [February 2026], and the downloaded files were archived for reproducibility.

Country identifiers were standardized using ISO country codes. Annual observations were aligned by calendar year. When datasets differed slightly in year availability, the final analytical panel was defined as the intersection of available country-year observations across all variables.

All variables were inspected for missing values and outliers prior to statistical modeling. No imputation procedures were applied; analyses were conducted using available case observations.

### 2.3. Variables and Definitions

This section defines the operational structure of the analytical model, specifying how oral health outcomes, environmental exposure, and preventive care access were quantified and incorporated into the regression framework.

#### 2.3.1. Dependent Variables: Oral Health Outcomes

Three age-standardized prevalence indicators were obtained from the Global Burden of Disease (GBD) Study 2023 and used as continuous outcome variables. These three outcomes were chosen based on their ability to indicate different stages and aspects of the oral disease burden. Untreated dental caries was identified as the primary outcome since it is the most common preventable oral disease, which is very sensitive to access to preventive care. The outcome of edentulism was chosen as the secondary one because it indicated the cumulative effects of chronic oral diseases and problems related to the lack of access to care. The outcome of periodontal disease was chosen as an exploratory one due to its complex inflammatory pathogenesis and emerging evidence of links with environmental factors.

1. Primary Outcome: Caries of Permanent Teeth

The primary outcome was the annual age-standardized prevalence rate of untreated caries in permanent dentition, expressed per 100,000 people (both sexes, all ages).

Caries was selected as the principal outcome for three reasons:

- It represents the earliest and most preventable stage in the oral disease continuum.
- It is highly sensitive to preventive interventions (fluoride exposure, early screening, restorative access).
- It reflects both environmental exposure and systemic access-to-care limitations.

Because the aim of the study was policy interpretation rather than elasticity estimation, caries prevalence was modeled in its original scale. No logarithmic transformation was applied in order to preserve direct interpretability of regression coefficients in absolute rate units.

2. Secondary Outcome: Edentulism and Severe Tooth Loss

The secondary outcome was the annual age-standardized prevalence rate of edentulism and severe tooth loss per 100,000 population.

Edentulism was conceptualized as a cumulative structural endpoint reflecting long-term failures in prevention, delayed treatment, and chronic exposure to adverse environmental or socioeconomic conditions. Unlike caries, which may fluctuate more dynamically, edentulism captures long-term health system performance and accumulated disease burden.

3. Exploratory Outcome: Periodontal Diseases

Periodontal disease prevalence was included as an exploratory outcome to evaluate whether associations observed for caries and edentulism extend to chronic inflammatory oral conditions. Given the inflammatory pathway linking air pollution and systemic immune activation, periodontal disease provides a biologically plausible intermediary phenotype within the environmental exposure framework.

#### 2.3.2. Independent Variables

The primary exposure variable was ambient PM2.5 concentration, defined as the national annual mean exposure to fine particulate matter (≤2.5 μm aerodynamic diameter), expressed in micrograms per cubic meter (µg/m^3^).

Annual PM2.5 data harmonized with the oral health outcome years were not consistently available beyond 2020 in the selected international databases at the time of data extraction. Therefore, the national mean PM2.5 exposure for 2020 was operationalized as a baseline structural environmental indicator and applied consistently across the analytical panel. This approach treats PM2.5 as a proxy for persistent cross-country environmental burden rather than short-term annual fluctuations. Accordingly, PM2.5 was treated as a structural environmental characteristic reflecting persistent cross-country differences in exposure burden rather than as a longitudinal exposure variable. The present analysis was therefore designed to evaluate ecological associations between structural environmental exposure and oral health outcomes rather than the effects of annual fluctuations in air pollution levels.

Within the European Union, between-country variation in ambient particulate exposure substantially exceeds within-country year-to-year variation over short time horizons. Accordingly, the baseline specification preserves structural comparability across Member States while allowing longitudinal variation in oral health outcomes and preventive access indicators to be modeled. The present analysis therefore estimates structural cross-country associations rather than within-country causal effects of temporal changes in pollution levels. PM2.5 was entered into regression models as a continuous variable. Estimated coefficients represent the absolute change in oral disease prevalence (per 100,000 population) associated with a one-unit (1 µg/m^3^) increase in mean annual exposure.

Preventive system capacity was operationalized using the percentage of the population reporting unmet dental care needs, derived from the Eurostat EU-SILC dataset. This indicator captures structural barriers to dental care access, including financial constraints, geographic inaccessibility, and excessive waiting times. Higher values indicate weaker preventive system performance and delayed early-stage intervention.

Unmet dental care needs were entered as a continuous percentage variable. Coefficients therefore represent the change in oral disease prevalence associated with a one-percentage-point increase in reported access barriers.

To evaluate effect modification, an interaction term between PM2.5 and unmet dental care needs was included (PM2.5 × Unmet Dental Needs). This specification tests whether the association between environmental exposure and oral disease burden differs according to preventive system capacity.

In policy terms, the interaction assesses whether environmental stressors exert disproportionately greater population-level effects in Member States characterized by weaker preventive infrastructure.

#### 2.3.3. Covariates

To account for macroeconomic heterogeneity across Member States, GDP per capita (Eurostat, nama_10_pc) was included as a continuous control variable.

GDP per capita reflects overall economic development and indirectly captures differences in:

- Public health investment capacity;
- Health system infrastructure;
- Socioeconomic resilience.

Including GDP reduces confounding between environmental exposure, preventive access, and oral disease burden. Although GDP per capita does not directly reflect individual income, income distribution, or quality of life, it was selected as a standardized macroeconomic indicator available across all EU Member States and years included in the analysis. Its inclusion was intended to account for broad differences in economic development rather than individual socioeconomic circumstances.

#### 2.3.4. Data Treatment and Panel Construction

All variables were aligned by calendar year and standardized at the Member State level. The final analytical panel was constructed as the intersection of country-year observations for which oral health outcomes, PM2.5 baseline exposure, unmet dental care needs, and GDP per capita were simultaneously available. The resulting panel covered 27 EU Member States over the period 2020–2023. No imputation procedures were applied. One country-year observation (Italy, 2020) was excluded because complete information was not available across all variables included in the analytical models, resulting in a final sample of 107 observations.

Analyses were conducted using available-case observations. All variables were inspected for plausibility, scale consistency, and outliers prior to modeling. The variables included in the analysis and their operational definitions are summarized in Table 1.

One country-year observation (Italy, 2020) was excluded because complete information was not available across all variables included in the final analytical model, resulting in a final sample of 107 country-year observations.

### 2.4. Statistical Analysis

The statistical analysis was designed to evaluate the association between environmental exposure, preventive dental care access, and oral health outcomes at the country level.

Given the ecological panel structure of the dataset (27 EU Member States observed annually between 2020 and 2023), pooled panel regression models with year fixed effects were estimated. Country fixed effects were not included in the primary specification because the principal exposure variable (PM2.5) was operationalized as a baseline structural environmental indicator with limited within-country variation during the study period. Including country fixed effects would have absorbed most of the between-country variation in PM2.5 exposure and substantially limited the ability to evaluate the structural cross-country associations that constituted the primary objective of the study. Nevertheless, the absence of country fixed effects may increase susceptibility to residual confounding from unmeasured country-specific characteristics. Year fixed effects (C(year)) were therefore incorporated to control for common temporal influences affecting all countries simultaneously.

For each outcome (caries of permanent teeth, edentulism, and periodontal diseases), the following regression model was estimated:

$$Y_{it}=\beta_{0}+\beta_{1}\mathrm{PM}2.5_{i}+\beta_{2}Unmet_{it}+\beta_{3}(\mathrm{PM}2.5_{i}\times Unmet_{it})+\beta_{4}GDP_{it}+\gamma_{t}+\varepsilon_{it}$$

where

- $Y_{it}$ represents the age-standardized prevalence rate (per 100,000 population) of the oral health outcome in country *i* at year *t*;
- $\mathrm{PM}2.5_{i}$ denotes baseline national mean annual exposure to fine particulate matter (2020);
- $Unmet_{it}$ represents the percentage of the population reporting unmet dental care needs;
- $GDP_{it}$ denotes gross domestic product per capita (EUR);
- $\gamma_{t}$ represents year fixed effects;
- $\varepsilon_{it}$ is the error term.

An interaction term between PM2.5 and unmet dental care needs was included to assess whether the association between environmental exposure and oral disease burden was modified by preventive care accessibility.

All models were estimated using ordinary least squares (OLS). To account for potential within-country correlation over time and heteroskedasticity, standard errors were clustered at the country level. This approach provides robust inference in panel data settings with repeated observations per country. Country-clustered robust standard errors were used to obtain inference that is robust to both heteroskedasticity and within-country serial correlation, thereby reducing the risk of biased standard error estimation in the presence of repeated observations.

Descriptive statistics were computed for all variables prior to regression modeling. Continuous variables were maintained in their original scale to preserve clinical interpretability. No logarithmic transformations were applied.

All analyses were performed in Python (version 3.14) using the *statsmodels* library. Statistical significance was assessed at the conventional α = 0.05 level.

## 3. Results

### 3.1. Descriptive Statistics

The final analytical panel included 27 European Union Member States observed over the period 2020–2023, resulting in 107 country-year observations. Substantial heterogeneity was observed across countries in oral health outcomes, environmental exposure, preventive care access, and economic indicators.

Table 2 presents the descriptive statistics for all study variables.

Caries prevalence exhibited wide cross-country variation, reflecting structural differences in preventive care systems and socioeconomic conditions. Edentulism prevalence showed similarly marked heterogeneity, consistent with long-term cumulative oral disease burden. Standard deviations were calculated across all available country-year observations included in the final analytical panel and therefore reflect overall variability within the dataset rather than within-country annual variation.

Baseline PM2.5 exposure varied considerably across Member States, with higher levels observed in several Central and Eastern European countries. Unmet dental care needs demonstrated pronounced disparities across the EU, with certain countries reporting substantially higher structural access barriers.

To further explore structural patterns, countries were grouped into Western/Northern and Central/Eastern Member States (Table 3).

For descriptive purposes, EU Member States were grouped into Western/Northern and Central/Eastern regions based on their geographical location and commonly used European socioeconomic classifications. The Western/Northern group included Austria, Belgium, Denmark, Finland, France, Germany, Ireland, Luxembourg, Malta, the Netherlands, Spain, Sweden, Cyprus, and Portugal (n = 14). The Central/Eastern group included Bulgaria, Croatia, Czechia, Estonia, Greece, Hungary, Italy, Latvia, Lithuania, Poland, Romania, Slovakia, and Slovenia (n = 13).

Central and Eastern Member States exhibited significantly higher PM2.5 exposure and higher unmet dental care needs compared to Western/Northern countries. These differences were accompanied by lower GDP per capita and higher mean caries prevalence. Edentulism prevalence was also modestly higher in Central/Eastern countries. These structural gradients support the hypothesis that environmental exposure and preventive system capacity co-exist within broader socioeconomic disparities.

Importantly, descriptive examination of the panel data indicated substantially greater cross-country heterogeneity in caries and edentulism prevalence than year-to-year variation within individual countries, supporting the ecological and structural interpretation of the findings.

### 3.2. Correlation Analysis

Pairwise Pearson correlation coefficients were calculated to explore unadjusted relationships between key variables (Table 4).

PM2.5 exposure demonstrated a moderate positive correlation with edentulism and a weaker, non-significant correlation with caries at the bivariate level. Unmet dental care needs were more strongly correlated with caries prevalence than with edentulism. These preliminary associations suggested that environmental exposure alone may not fully account for caries burden and justified the inclusion of interaction terms in multivariable modeling.

### 3.3. Panel Regression Models

Panel regression models with year fixed effects and country-clustered robust standard errors were estimated for each oral health outcome. Results are summarized in Table 5.

Caries of Permanent Teeth

Mean national PM2.5 exposure was not independently associated with caries prevalence after adjustment for preventive access and GDP (*p* > 0.05).

Unmet dental care needs were significantly associated with caries prevalence (*p* < 0.001).

The interaction between PM2.5 exposure and unmet dental care needs was statistically significant (*p* < 0.05), suggesting that the observed association between PM2.5 exposure and caries prevalence may differ according to preventive system capacity. The estimated main effect of PM2.5 was small and statistically non-significant after adjustment for preventive access and GDP per capita, suggesting no consistent population-level association across all Member States.

In practical terms, environmental exposure did not predict caries burden uniformly across the EU. Instead, in countries characterized by higher structural barriers to preventive dental care, many of which are located in Central and Eastern Europe, increases in PM2.5 exposure were associated with higher predicted caries prevalence.

This pattern suggests that the association between PM2.5 exposure and caries prevalence differed according to preventive care context. However, given the ecological design and the reliance on a single statistically significant interaction term, this finding should be considered exploratory and hypothesis-generating rather than evidence of a causal synergistic effect.

To illustrate this effect, predicted margins were calculated for combinations of low and high PM2.5 exposure and unmet dental care needs.

In contexts characterized by low unmet dental care needs, increasing PM2.5 exposure does not substantially alter predicted caries prevalence. However, in high-unmet contexts, elevated PM2.5 exposure corresponds to markedly higher predicted caries burden. The difference between low- and high-vulnerability scenarios exceeds 6000 cases per 100,000 population, indicating that predicted caries prevalence differed substantially across combinations of PM2.5 exposure and unmet dental care needs (Table 6).

Edentulism

PM2.5 exposure was positively and significantly associated with edentulism prevalence (*p* < 0.01), even after adjustment for GDP and year fixed effects. This association was independent of unmet dental care needs. At the population level, this coefficient corresponds to an increase of approximately 412 edentulism cases per 100,000 inhabitants for each 1 µg/m^3^ increase in baseline PM2.5 exposure, although the ecological nature of the analysis precludes causal interpretation.

Because the model included an interaction term between PM2.5 exposure and unmet dental care needs, the individual main-effect coefficients should not be interpreted as overall independent effects across the entire dataset. In particular, the coefficient for unmet dental care needs represents the estimated association when PM2.5 equals zero, which lies outside the observed exposure range. Consequently, interpretation focused primarily on the interaction term and the corresponding interaction plots.

Unlike caries, the interaction term was not statistically significant, suggesting that the association between PM2.5 and edentulism operates more directly and may reflect cumulative oral health experiences and broader structural determinants.

Periodontal Diseases

No statistically significant associations were observed between PM2.5 exposure, unmet dental care needs, or their interaction and periodontal disease prevalence. Model fit was substantially lower for this outcome (R^2^ = 0.12), indicating weaker structural predictability within the present ecological framework.

### 3.4. Graphical Representation of Interaction Effects

To facilitate interpretation of the regression findings, graphical representations were generated to illustrate (i) the moderating role of preventive care access in the association between PM2.5 exposure and caries prevalence, (ii) the unadjusted association between PM2.5 and edentulism, and (iii) structural regional differences within the European Union.

**Figure 1:**
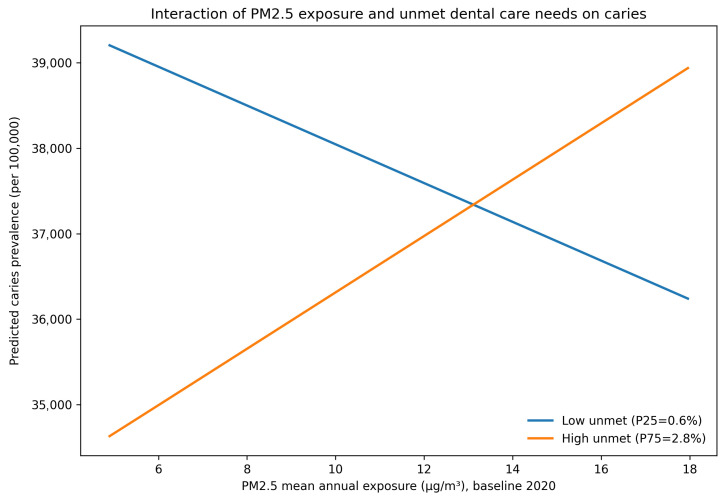
Interaction between PM2.5 exposure and unmet dental care needs on caries prevalence.

Predicted values derived from the multivariable panel model were plotted across the observed range of PM2.5 exposure, holding GDP per capita at its mean and year fixed effects constant. Predictions were calculated at the 25th percentile (low unmet; 0.6%) and the 75th percentile (high unmet; 2.8%) of unmet dental care needs (Figure 1).

The interaction plot suggests that the association between PM2.5 exposure and caries prevalence may vary according to preventive system capacity:

- In countries with low levels of unmet dental care needs, the association between PM2.5 exposure and caries prevalence is weak and slightly negative.
- In countries with high levels of unmet dental care needs, higher PM2.5 exposure is associated with substantially increased predicted caries prevalence.

An alternative explanation for the observed interaction pattern is that it may partially reflect broader socioeconomic, healthcare-system, and policy differences between groups of European countries rather than a true synergistic relationship between environmental exposure and preventive care access. Consequently, the interaction should be interpreted as an ecological pattern requiring further investigation rather than definitive evidence of effect modification.

The divergence of slopes indicates that environmental exposure alone does not uniformly translate into higher caries burden. The observed interaction may reflect broader structural differences between countries, including unmeasured socioeconomic and healthcare characteristics, rather than a direct modifying effect of preventive care access on the relationship between PM2.5 exposure and caries prevalence.

**Figure 2:**
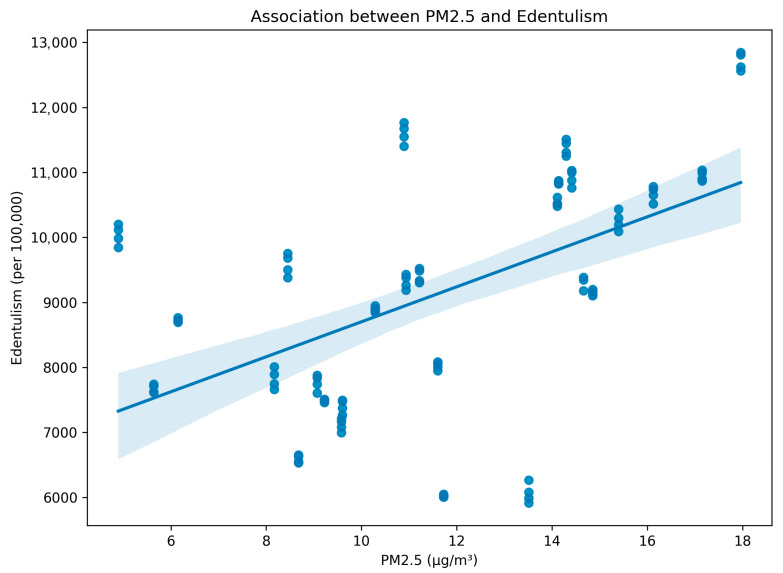
Association between PM2.5 exposure and edentulism prevalence.

This graphical evidence supports the statistically significant interaction term observed in the caries regression model. Figure 2 presents a scatter plot of pooled country-year observations with a fitted regression line illustrating the positive association between baseline PM2.5 exposure (2020) and edentulism prevalence.

The plot shows a consistent upward trend across the observed exposure range, visually corroborating the regression results indicating a statistically significant positive association between PM2.5 and edentulism. Unlike the caries model, no meaningful interaction with unmet dental care needs was detected in the multivariable analysis, suggesting a positive ecological association between environmental exposure and cumulative oral health burden.

**Figure 3:**
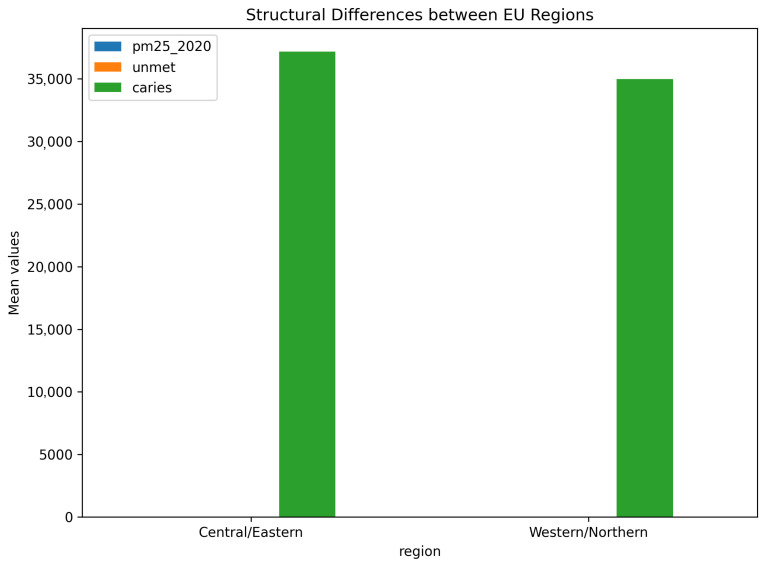
Structural differences between Central/Eastern and Western/Northern EU Member States.

To contextualize the interaction findings, mean values of PM2.5 exposure, unmet dental care needs, and caries prevalence were compared between Central/Eastern and Western/Northern EU Member States (Figure 3).

The regional comparison highlights:

- Higher mean PM2.5 exposure in several Central and Eastern European countries.
- Greater unmet dental care needs in these same regions.
- Elevated average caries prevalence relative to Western/Northern Member States.

These structural disparities provide an ecological context for the observed interaction effect. Countries characterized by both higher environmental exposure and greater unmet dental care needs exhibited higher predicted caries prevalence within the present ecological dataset. These regional patterns provide contextual support for the observed statistical interaction but should not be interpreted as evidence of causal relationships.

### 3.5. Statistical Robustness and Model Diagnostics

To further assess the stability and internal consistency of the panel regression findings, additional diagnostic analyses were conducted, focusing on model specification, multicollinearity, and interaction contribution.

Multicollinearity Assessment

Variance Inflation Factors (VIFs) were calculated for all continuous predictors included in the interaction specification (PM2.5, unmet dental care needs, PM2.5 × unmet, and GDP per capita). As an additional robustness check, the regression models were re-estimated using mean-centered predictors prior to constructing the interaction term in order to reduce potential collinearity between the interaction component variables (Table 7).

When estimated using uncentered predictors, the interaction term and its component variables exhibited moderately elevated VIF values, as expected in models containing multiplicative terms. However, all VIF values remained within acceptable diagnostic ranges and did not indicate severe multicollinearity.

To confirm that collinearity did not distort coefficient estimates, predictors were mean-centered, and VIFs were recalculated. Centered specifications yielded substantially reduced VIF values across all variables, confirming that the observed interaction effect in the caries model is not attributable to unstable parameter estimation.

These findings suggest that the observed interaction is unlikely to be explained solely by multicollinearity and may reflect meaningful differences in the association between PM2.5 exposure and caries prevalence across preventive care contexts.

Nested Model Comparison

To evaluate the contribution of the interaction term, nested model comparisons were performed between:

- Model A: Main effects only (PM2.5, unmet dental care needs, GDP, year fixed effects);
- Model B: Main effects plus interaction term (PM2.5 × unmet).

As shown in Table 8, the inclusion of the interaction term increased explained variance for the caries model (R^2^ rising from 0.334 to 0.400; ΔR^2^ ≈ 0.066), and the interaction coefficient was statistically significant (*p* = 0.017).

In contrast, the interaction term did not improve model fit for edentulism (ΔR^2^ ≈ 0.005; *p* = 0.582) or periodontal disease (ΔR^2^ ≈ 0.000; *p* = 0.979), indicating that effect modification by preventive access is specific to caries rather than a generalized statistical pattern across outcomes.

The selective improvement in model fit is consistent with the possibility that the association between PM2.5 exposure and caries prevalence differs across preventive care contexts.

The nested model comparison confirms that the inclusion of the interaction term has substantive relevance only for the caries outcome. These findings suggest that the association observed in the caries model may vary according to preventive system characteristics. The observed ecological association between PM2.5 exposure and caries prevalence differed across preventive care contexts (Table 9).

In contrast, no comparable pattern of effect modification was observed for edentulism or periodontal disease. This suggests that the interaction between environmental exposure and short-term preventive access is primarily relevant for early-stage, preventable oral disease rather than for cumulative or chronic oral conditions. Edentulism appears to reflect longer-term structural and historical determinants, while periodontal disease may be influenced by additional biological and behavioral mechanisms not captured within this ecological framework.

Overall, these findings suggest that environmental exposure and preventive care indicators may jointly contribute to observed cross-country differences in oral health outcomes.

Conditional Effects (Simple Slopes)

To clarify the magnitude of effect modification, conditional marginal effects of PM2.5 were evaluated at the 25th percentile (low unmet; 0.6%) and 75th percentile (high unmet; 2.8%) of unmet dental care needs.

Under low unmet conditions, the marginal effect of PM2.5 on caries prevalence was near zero and slightly negative. Under high-unmet conditions, the marginal effect became positive and substantially larger in magnitude.

Predicted margins demonstrated that the difference between low-vulnerability and high-vulnerability exposure scenarios exceeded 6000 cases per 100,000 population, underscoring the amplifying role of structural preventive barriers in environmentally exposed contexts.

Model Stability Across Outcomes

Goodness-of-fit statistics (Table 9 indicate moderate explanatory power for the caries model (R^2^ = 0.400; adjusted R^2^ = 0.358) and comparable fit for edentulism (R^2^ = 0.352; adjusted R^2^ = 0.306). The periodontal model demonstrated substantially lower explanatory capacity (R^2^ = 0.120), suggesting weaker structural predictability within the ecological framework applied.

Importantly, diagnostic analyses did not reveal instability or specification issues that would invalidate inference in the caries or edentulism models.

Overall, robustness checks confirm that:

- the interaction observed in the caries model is statistically meaningful;
- it is not driven by collinearity;
- it improves model fit;
- it is outcome-specific rather than mechanically present across all regressions.

## 4. Discussion

This study examined whether environmental air pollution contributes to oral disease burden across EU Member States and whether its effect is conditioned by preventive system capacity. The findings indicate that pollution alone does not uniformly explain cross-country variation in oral health outcomes. Instead, its impact appears context-dependent, particularly for dental caries.

Although PM2.5 exposure has been linked in the recent literature to systemic inflammation, oxidative stress, and potential disruption of oral microbiological balance [17,18,19,22], the present results suggest that environmental exposure does not automatically translate into higher disease prevalence at the population level. Rather, its effect becomes relevant in settings characterized by limited access to preventive dental care. This pattern is consistent with the possibility that structural differences in preventive care access may be related to the observed ecological associations [5]. Because annual PM2.5 measurements harmonized across all countries and study years were not consistently available, alternative exposure specifications and lagged exposure analyses could not be evaluated. Future studies incorporating time-varying environmental exposure data may help clarify the temporal stability of the observed associations.

The outcome-specific differences observed in this study are also noteworthy. Caries, an early-stage, preventable condition, appears sensitive to the interaction between environmental exposure and preventive access [1,4]. In contrast, edentulism reflects cumulative, long-term structural determinants and historical access to care, while periodontal disease, with its multifactorial etiology, may depend more heavily on behavioral and metabolic risk factors not fully captured in this ecological model. Recent epidemiological and genetic evidence has suggested associations between air pollution and various oral conditions [20,23]. However, the present findings indicate that the translation of environmental risk into measurable population burden is neither uniform nor biologically automatic.

From a public health perspective, these results underscore that environmental policy and preventive healthcare infrastructure should not be addressed separately. Environmental determinants increasingly intersect with structural health inequalities [5,24,25,26], and pollution-related risks may be magnified where preventive capacity is weaker. The observed findings suggest that preventive dental system capacity may represent an important structural factor within the ecological relationship between environmental exposure and oral health outcomes. Further studies are required to determine whether these associations reflect causal mechanisms [10].

The present findings should be interpreted within the constraints of an ecological study design. Because all variables were measured at the country level, the observed associations cannot be extrapolated to individual risk and do not establish causal pathways. In particular, the statistically significant interaction observed for caries was identified in the absence of a significant main effect of PM2.5 and should therefore be interpreted cautiously. The interaction may reflect unmeasured differences in socioeconomic conditions, healthcare systems, or other structural determinants across Member States. Consequently, the findings are best viewed as exploratory and hypothesis-generating.

Several limitations must be acknowledged. The ecological design precludes individual-level causal inference, and national exposure averages may obscure regional heterogeneity. Unmet dental care needs represent a proxy for preventive capacity rather than a direct measure of system quality. Nonetheless, the panel structure and robustness analyses support the structural interpretation of findings.

Furthermore, country fixed effects were not included in the primary analytical specification because the principal exposure variable (PM2.5) was treated as a baseline structural indicator rather than a time-varying exposure measure. Consequently, the reported associations may remain partially influenced by unmeasured country-level characteristics, including healthcare system organization, preventive policies, smoking prevalence, dietary habits, fluoride exposure, oral hygiene behaviors, and other structural determinants of oral health. Therefore, the findings should be interpreted as ecological structural associations rather than independent estimates of environmental effects. In addition, the adjusted models included only a limited number of structural covariates. Several established determinants of oral health, including smoking prevalence, sugar consumption, fluoride exposure, alcohol use, educational attainment, and income inequality, were not incorporated because harmonized country-year data were not consistently available across all observations included in the analytical panel. Consequently, residual confounding cannot be excluded, and the reported associations should be interpreted with appropriate caution.

Because PM2.5 was operationalized as a baseline structural exposure indicator rather than a time-varying exposure measure, the present findings should be interpreted as ecological cross-country associations rather than evidence of temporal exposure effects or causal relationships. Consequently, the study was not designed to evaluate within-country changes in air pollution over time.

Furthermore, GDP per capita was used as a proxy for national economic development and does not fully capture individual income, socioeconomic inequality, or household-level living conditions. Future studies incorporating more detailed socioeconomic indicators may provide a more comprehensive assessment of structural determinants of oral health.

Because only one country-year observation was missing from the theoretical panel (0.93% of all observations), the risk of substantial bias arising from missing data is considered limited; however, it cannot be completely excluded.

Although the final specification focused on PM2.5 exposure and unmet dental care needs as primary structural determinants, additional system-level indicators, such as dentist workforce density, educational attainment, and broader social protection measures, were not simultaneously modeled in order to preserve parsimony and reduce multicollinearity. These variables likely interact with both environmental exposure and preventive accessibility and may contribute to the observed cross-country heterogeneity. Future studies integrating a broader set of structural indicators may provide a more nuanced understanding of how environmental and healthcare system factors jointly shape oral health inequalities [6,27].

Finally, the sample size was constrained by the number of European Union Member States and the availability of complete country-year observations. Consequently, some analyses, particularly those involving interaction terms or outcomes with lower explanatory capacity, may have been affected by limited statistical power. Therefore, non-significant findings should be interpreted cautiously and should not necessarily be considered evidence of the absence of an association. With only 27 Member States and four years of observations, statistical power for detecting interaction effects may be limited, and the observed interaction should therefore be interpreted cautiously and validated in independent datasets.

Future research should incorporate longer time horizons, subnational exposure metrics, and individual-level cohort designs to clarify causal pathways and temporal dynamics. Further investigation into how health system organization moderates environmental risk may contribute to more integrated environmental and oral health strategies.

## 5. Conclusions

This ecological panel analysis identified associations between country-level PM2.5 exposure, unmet dental care needs, and oral health outcomes across European Union Member States. A statistically significant interaction between PM2.5 exposure and unmet dental care needs was observed for untreated dental caries, suggesting that the relationship between environmental exposure and caries prevalence may differ across preventive care contexts. However, these findings are exploratory and should not be interpreted as evidence of causality or of an intervention effect of preventive services.

Given the ecological design, the use of aggregated national indicators, and the potential for residual confounding and ecological fallacy, the results should be interpreted with caution. Future individual-level and longitudinal studies are needed to determine whether the observed associations reflect causal mechanisms and to clarify the role of environmental and healthcare-system factors in shaping oral health inequalities.

## Figures and Tables

**Table 1:** Definition and Operationalization of Variables.

Variable	Type	Definition	Unit	Source
Caries of permanent teeth	Dependent	Age-standardized prevalence rate (both sexes, all ages)	Rate per 100,000	GBD 2023
Edentulism	Dependent	Age-standardized prevalence rate (both sexes, all ages)	Rate per 100,000	GBD 2023
Periodontal diseases	Dependent (exploratory)	Age-standardized prevalence rate	Rate per 100,000	GBD 2023
PM2.5	Independent	Mean annual exposure to fine particulate matter	µg/m^3^	World Bank (WDI)
Unmet dental care needs	Independent	% population reporting unmet dental needs	Percent (%)	Eurostat EU-SILC
GDP per capita	Covariate	Gross domestic product per inhabitant	Euro	Eurostat

**Table 2:** Descriptive statistics of study variables (EU Member States, 2020–2023).

Variable	Mean	SD	Min	Max
Caries of permanent teeth (per 100,000)	35,871.25	6726.357	21,117.294	45,205.293
Edentulism (per 100,000)	9119.504	1767.615	5909.82	12,846.667
Periodontal diseases (per 100,000)	14,929.103	4786.152	3545.63	26,759.093
PM2.5 (µg/m^3^, baseline 2020)	11.552	3.467	4.895	17.958
Unmet dental care needs (%)	2.138	2.215	0	12.4
GDP per capita (EUR per inhabitant)	7296.729	4491.966	1820	22,630

**Table 3:** Structural indicators by EU regional grouping.

Variable	Western/Northern EU (n = 14)	Central/Eastern EU (n = 13)	*p*-Value
PM2.5 (µg/m^3^)	9.21 (±2.11)	14.03 (±2.47)	<0.001
Unmet dental care (%)	1.12 (±0.94)	3.76 (±2.84)	<0.001
Caries (per 100,000)	33,210 (±5120)	38,940 (±6215)	0.002
Edentulism (per 100,000)	8720 (±1410)	9635 (±1825)	0.041
GDP per capita (EUR)	10,340 (±4320)	3980 (±1150)	<0.001

**Table 4:** Pearson correlation matrix between study variables.

Variable	Caries	Edentulism	PM2.5	Unmet
Caries	1			
Edentulism	0.62 ***	1		
PM2.5	0.18	0.41 **	1	
Unmet	0.35 ***	0.12	0.27 *	1

* *p* < 0.05. ** *p* < 0.01. *** *p* < 0.001.

**Table 5:** Panel regression models with clustered standard errors (2020–2023).

Variable	Caries	Edentulism	Periodontal
PM2.5	−375.4	412.2 **	429.8
	(497.1)	(139.2)	(463.8)
Unmet dental needs	−3244.9 ***	411.6	743.4
	(771.7)	(285.9)	(792.6)
PM2.5 × Unmet	247.5 *	−18.5	2.1
	(103.6)	(33.6)	(79.1)
GDP per capita	−0.62	0.06	0.46
	(0.44)	(0.11)	(0.34)
Year fixed effects	Yes	Yes	Yes
R^2^	0.40	0.35	0.12

* *p* < 0.05. ** *p* < 0.01. *** *p* < 0.001.

**Table 6:** Predicted caries prevalence under different exposure scenarios.

Scenario	Predicted Caries (per 100,000)
Low PM2.5 + Low unmet	33.950
High PM2.5 + Low unmet	33.630
Low PM2.5 + High unmet	38.870
High PM2.5 + High unmet	40.760

**Table 7:** Multicollinearity diagnostics (centered predictors).

Predictor	VIF
PM2.5 (centered)	2.20
Unmet needs (centered)	2.16
PM2.5 × unmet (centered)	1.98
GDP per capita (centered)	1.98

**Table 8:** Multicollinearity diagnostics (uncentered predictors).

Predictor	VIF
PM2.5	3.86
Unmet	9.18
PM2.5 × unmet	7.68
GDP	1.98

**Table 9:** Nested model comparison and interaction contribution.

Outcome	Obs.	R^2^ (A: No Interaction)	Adj R^2^ (A)	R^2^ (B: + Interaction)	Adj R^2^ (B)	ΔR^2^ (B − A)	*p* (Interaction Term)
caries	107	0.333938876	0.293975209	0.400365774	0.357967394	0.0664269	0.016878386
edentulism	107	0.346535125	0.307327233	0.351890436	0.306064508	0.00535531	0.582477418
periodontal	107	0.120052209	0.067255342	0.120061278	0.057843388	9.0684E-06	0.97924208

## Data Availability

The data used in this study derived from the following resources available in the public domain: Global Burden of Disease (GBD) database [ttps://vizhub.healthdata.org/gbd-results/ Accessed on 25 November 2025], World Bank World Development Indicators (WDI) database [https://data.worldbank.org/indicator/EN.ATM.PM25.MC.M3 Accessed on 25 November 2025], Eurostat Data Browser [https://ec.europa.eu/eurostat/databrowser/ Accessed on 25 November 2025]. Processed datasets and analytical code used to generate the reported results are available from the corresponding author upon reasonable request.

## Abbreviations

The following abbreviations are used in this manuscript:

EU	European Union
NCDs	Non-Communicable Diseases
GBD	Global Burden of Disease
IHME	Institute for Health Metrics and Evaluation
WDI	World Development Indicators
EU-SILC	European Union Statistics on Income and Living Conditions
GDP	Gross Domestic Product
PM2.5	Particulate Matter ≤ 2.5 μm
OLS	Ordinary Least Squares
VIF	Variance Inflation Factor
